# Coda: the experience of war beyond exceptionalism

**DOI:** 10.1080/07292473.2024.2409534

**Published:** 2024-10-14

**Authors:** Antonio De Lauri

**Affiliations:** Chr. Michelsen Institute, Bergen, Norway

**Keywords:** war, fun, exceptionalism, soldiers, fighters

## Abstract

When linked to the context of war, fun can be understood as an expression of both direct and indirect communication, a manner of public engagement as well as a ‘ritual of inversion’ in which the proprieties of structure (the declared mandate and rules of war) are lampooned and violated, yet the finalities of the project of war (dominion, control, violence, and so on) remain intact. The focus on fun is not meant to trivialise the suffering war produces. On the contrary, it encourages a more honest and accurate analysis of what actively experiencing war entails. There are different reasons for pursuing a line of research that delves into the articulation of different emotions, moralities, and fighters’ perspectives, for instance the need to de-exceptionalise war’s brutality.

The pace of the increase in global military expenditure is a telling indication of the armed governance that characterises geopolitics and international relations.^1^ In the past few decades alone, millions of lives have been claimed by wars waged by imperial powers such as the United States, Russia, and the United Kingdom, and by conflicts and unrest in contexts such as Darfur, Myanmar, Kivu, or Yemen. Of course, the immediate scale and intensity of a specific conflict are not the only elements to define the long-term tragedy that war generates. The reverberating effects of the United States’ War on Terror, for example, have been devastating. According to the Costs of War project:

at least 940,000 people have been killed by direct war violence in Iraq, Afghanistan, Syria, Yemen, and Pakistan. The number of people who have been wounded or have fallen ill as a result of the conflicts is far higher, as is the number of civilians who have died indirectly as a result of the destruction of hospitals and infrastructure and environmental contamination, among other war-related problems.^2^

The long-term effects of war are always difficult to measure, and loss of life certainly might exceed standard assessments. The bombing of Libya by the multi-state NATO-led coalition in 2011, for instance, produced widespread national and regional instability that, as of today, is far from being resolved.^3^ The military intervention was implemented under the auspices of United Nations Security Council Resolution 1973, proposed by France, Lebanon, and the United Kingdom (with the declared intention to *protect* civilians) and voted for by several Security Council members, including the United States, then under the administration of Nobel Peace Prize winner (!) Barack Obama (also remembered by Afghans for the massive bombing of Afghanistan). Consequences of war may also include the dismantling of broader ideas of law and justice. For instance, the ongoing genocidal devastation of Gaza by the Israeli military and government,^4^ with the direct and indirect support of many Western countries, is creating – beyond the enormous human suffering of the Palestinian people – a global crisis of credibility and legitimacy for the international legal system. Considering the complete lack of respect for international humanitarian law shown by Israel and its Western allies, the notion of ‘double standards’ is an empirical reality that can no longer be neglected.^5^

At the international level, war continues to represent a plausible modality of governance, at times with widespread political and public support. The current conflict in Ukraine (which can be divided into two phases, 2014–22 and 2022–present) has resuscitated, in its second phase, a certain dangerous fascination with war. Journalists, analysts, and politicians wearing real or symbolic military helmets have proliferated globally following the Russian invasion in 2022. Notions such as patriotism, defence of democratic values, the right side of history, or a new fight for freedom have been mobilised as imperatives for everyone to take a side in this war. It is not surprising, then, that a large number of so-called foreign fighters have been willing to go to Ukraine to join one side or the other. I met a few of them in 2022 at the Poland–Ukraine border, where I was conducting interviews, along with a Norwegian film crew, with soldiers and foreign fighters who were either entering or exiting the war zone. Some of them actually never got to fight or be recruited as they lacked military experience or appropriate motivation. The people we met had different backgrounds. Some of them had spent years in the military, while others only did military service. Some had family at home waiting for them; others had no home to go back to. Some had strong ideological motivations; others were just willing to shoot at something or someone.^6^

There was also a large group of former soldiers who had transitioned towards ‘humanitarian work’. As we were crossing the border to get into Ukraine, a former US soldier told us: ‘The reason why many retired or former soldiers moved to humanitarian work might easily be the need for excitement’. Once you leave the military, the activity that can take you closest to the ‘fun zone’, as another former US soldier said, referring to the war zone in Ukraine, is humanitarian work – or, in fact, a series of other businesses mushrooming in the proximity of war, including contractors and criminal activities. ‘We are adrenaline junkies’, the former US soldier told us.

What many of the foreign fighters had in common was the need to find a purpose in life as well as to search for excitement. K, a boy from Scandinavia in his early twenties who decided to join the legion of foreign fighters, believed that ‘being there’ was the right thing to do. He was willing to die and to kill. At the same time, he believed that it was an exciting experience and said at least one-third of the foreign fighters he had met were there to have fun. The category of ‘fun’ appears to a large extent as an oxymoron when situated in war. And yet in the stories of soldiers and veterans we find regular reference to ideas such as joy, excitement, allure, and fun. The former US soldier mentioned above said ‘we would be over-joyous’ after a military operation. A former military official I interviewed in Italy told me that being in a combat zone is thrilling, and that ‘you can experience fun, at times with a sense of guilt’.

This special issue of *War and Society* provides empirical and historical material to nuance the understanding of participation in war by addressing its dynamic emotional, experiential, and moral articulations, beyond more normative and doctrinal interpretations. Using ‘fun’ as an entry point to explore these complexities, this special issue allows for the perspectives of those who actively participate in war to emerge without forcing them into rigid external categories. The meaning of fun is often taken for granted both in scientific literature and in everyday interactions; beyond dictionary definitions, there are few explanations of what fun involves and how to differentiate it from other social experiences. In the WARFUN project,^7^ which ignited the publication of this special issue, fun is understood as an expression of both direct and indirect communication, a manner of public engagement as well as a ‘ritual of inversion’ in which the proprieties of structure (the declared mandate and rules of war) are lampooned and violated, yet the finalities of the project of war (dominion, control, violence, and so on) remain intact. We do not refer here to the ‘mystical power of symbolic inversion in the ritual context’,^8^ but rather to the capacity of reiterated social experiences to mobilise symbolic and performative power. Indeed, the focus on fun is not meant to trivialise the suffering war produces. On the contrary, it encourages a more honest and accurate analysis of what actively experiencing war entails.

One striking element that emerges from this special issue is that soldiers and fighters are often more prone to talk about war for what it really is (in all its contradictions) as compared with (some) politicians, journalists, or academics. There are different reasons for pursuing a line of research that delves into the dynamics explored in this special issue. One reason certainly relates to the need to de-exceptionalise war’s brutality. For instance, there is a dominant propaganda that seems to suggest that war can be conducted according to a set of acceptable, standardised, and abstract rules. It puts forth an idea of a well-behaved war where only military targets are destroyed, force is not used in excess, and right and wrong are clearly defined. This rhetoric is used by governments, the mass media, and also scholars to make war more acceptable, even attractive, for the masses. Whatever deviates from this idea of a proper and noble war is considered an exception.^9^ US soldiers torturing prisoners in Abu Ghraib for fun: an exception. German soldiers playing with a human skull in Afghanistan: an exception. The US soldier who went on a house-to-house rampage in an Afghan village, killing sixteen civilians, including several children, for no reason: an exception. War crimes committed by Australian troops in Afghanistan: an exception. Iraqi prisoners tortured by British troops: an exception. Members of the Stryker Combat Brigade in Afghanistan accused of killing civilians for sport: an exception. French airstrikes at a wedding party in Mali: an exception. The Mahmudiyah rape and murders where US soldiers raped a fourteen-year-old girl and killed her and her family: an exception.

Many stories of soldiers torturing other soldiers or civilians and news about behaviour that far exceeds combat duties have emerged in the current war in Ukraine too, as well as in Gaza. To mention only one recent example, Israeli soldiers have been posting photos and videos of themselves toying with women’s lingerie found in Palestinian homes.^10^ Contrary to what some academics believe,^11^ that is to say, that the gravity of war (and especially some specific wars) is inconsistent with ‘fun’, we are overwhelmed by examples showing that even in what many have described as a live-streamed genocide, we find instances of fun (again, understood in all its complexity, including its darkest side).

All exceptions? No. This is exactly what war is. Governments make extensive efforts to explain that these kinds of episodes don’t belong to a *normal war* conducted according to International Humanitarian Law, reiterating the idea of the possibility of a decent war without any excess or extravagance. It is a war that only exists in theory. In the narrative of the good and decent war, even the killing of civilians is recounted with hypocrisy as a side effect, even though systematically targeting civilians is a feature of all contemporary wars (Gaza, again, is a clear example). Those with direct experience of war know well that the idea of a clean and efficient war is a lie. War is a chaotic universe of military strategies and actions intertwined with inhumanity, violations, uncertainty, doubts, and deceit. In all combat zones emotions such as fear, shame, joy, excitement, surprise, anger, cruelty, and compassion coexist. Together, these emotions shape the way war permeates the memories and bodies of those who experience it. The WARFUN project and this special issue illustrate once again that the complexity of human behaviour cannot be oversimplified in abstract terms. What happens on the terrain of war mirrors the most extreme aspects of resilience (for example fun as a mechanism to cope with war atrocities) or sadism (for example torture as fun). To be sure, whereas this is something we can observe among those who participate in war, these extremes relate to collective societal patterns that can be found outside the realm of war. Clearly, the responsibility for what happens in war rests not only with those who hold the gun, but also with those who make war possible at different distances from the combat area.

## Footnotes

1 SIPRI’s Military Expenditure Database, <https://www.sipri.org/databases/milex> [accessed April 29, 2024].

2 ‘Costs of War’, Watson Institute of International & Public Affairs, Brown University, <https://watson.brown.edu/costsofwar/costs/human> [accessed May 8, 2024].

3 Irene Costantini, ‘Conflict Dynamics in Post-2011 Libya: A Political Economy Perspective’, *Conflict, Security and Development* 16, no. 5 (2016), 405–22; Amnesty International, ‘Libya: The Forgotten Victims of NATO Strikes’, March 2012, <https://www.amnesty.org/en/wp-content/uploads/2021/07/mde190032012en.pdf> [accessed May 8, 2024]; Chris Hedges, ‘Libya Dam Disaster Shows Horrific Consequences of US/NATO Imperialism’, *Common Dreams*, 18 September 2023, <https://www.commondreams.org/author/chris-hedges> [accessed May 8, 2024].

4 The Application of the Convention on the Prevention and Punishment of the Crime of Genocide in the Gaza Strip (South Africa v. Israel), <https://www.icj-cij.org/case/192> [accessed May 8, 2024]; Amnesty International, ‘UN Special Rapporteur Report on Gaza Provides Crucial Evidence that Must Spur International Action to Prevent Genocide’, 26 March 2024, <https://www.amnesty.org/en/latest/news/2024/03/un-special-rapporteur-report-on-gaza-provides-crucial-evidence-that-must-spur-international-action-to-prevent-genocide/> [accessed April 29, 2024]; Iqbal Jassat, ‘Global Outrage Is Growing against Netanyahu’s Genocide in Gaza’, *Middle East Monitor*, 29 April 2024, <https://www.middleeastmonitor.com/20240429-global-outrage-is-growing-against-netanyahus-genocide-in-gaza/> [accessed April 29, 2024].

5 Julie Billaud and Antonio De Lauri, ‘How South Africa Rescued Humanity (and International Law) at the International Court of Justice’, *Public Anthropologist blog*, 19 January 2024, <https://publicanthropologist.cmi.no/2024/01/19/how-south-africa-rescued-humanity-and-international-law-at-the-international-court-of-justice/> [accessed May 7, 2024].

6 Antonio De Lauri, ‘The Idea of a Clean and Efficient War Is a Dangerous Lie’, 8 April 2022, *Common Dreams*, <https://www.commondreams.org/views/2022/04/08/idea-clean-and-efficient-war-dangerous-lie> [accessed May 13, 2024].

7 ‘War and Fun: Reconceptualizing Warfare and Its Experience’ (WARFUN) is a European Research Council (ERC) Consolidator Grant project (Grant agreement 101001106), <https://www.cmi.no/projects/2535-erc-war-and-fun> [accessed May 8, 2024].

8 Jacob Pandian, ‘Symbolic Inversions: An Interpretation of Contrary Behavior in Ritual’, *Anthropos* 96 (2001), 557–62.

9 De Lauri, ‘The Idea of a Clean and Efficient War Is a Dangerous Lie’.

10 Belén Fernández, ‘Weaponising Underwear: Genocide with a Semi-pornographic Twist’, *Al Jazeera*, 12 April 2024, <https://www.aljazeera.com/opinions/2024/4/12/weaponising-underwear-genocide-with-a-semi-pornographic-twist> [accessed May 8, 2024].

11 In the context of the WARFUN project, we have encountered a few cases of ‘reluctance’ from academics towards the framework of the research. Interestingly, those who were reluctant were exclusively academics who study war via books but have never conducted fieldwork with combatants or in a war context (let alone experienced war themselves).

